# A new two-tier strength assessment approach to the diagnosis of weakness in intensive care: an observational study

**DOI:** 10.1186/s13054-015-0780-5

**Published:** 2015-02-26

**Authors:** Selina M Parry, Sue Berney, Catherine L Granger, Danielle L Dunlop, Laura Murphy, Doa El-Ansary, René Koopman, Linda Denehy

**Affiliations:** School of Health Sciences, Physiotherapy Department, The University of Melbourne, Level 7 Alan Gilbert Building, 161 Barry Street, Parkville, 3010 VIC Australia; Department of Physiotherapy, Austin Health, 145 Studley Road, Heidelberg, 3084 VIC Australia; Institute for Breathing and Sleep, Austin Health, 145 Studley Road, Heidelberg, 3084, VIC Australia; Department of Physiology, The University of Melbourne, Grattan Street, Parkville, 3010 VIC Australia

**Keywords:** ■■■

## Abstract

**Introduction:**

Intensive care unit-acquired weakness (ICU-AW) is a significant problem. There is currently widespread variability in the methods used for manual muscle testing and handgrip dynamometry (HGD) to diagnose ICU-AW. This study was conducted in two parts. The aims of this study were: to determine the inter-rater reliability and agreement of manual muscle strength testing using both isometric and through-range techniques using the Medical Research Council sum score and a new four-point scale, and to examine the validity of HGD and determine a cutoff score for the diagnosis of ICU-AW for the new four-point scale.

**Methods:**

Part one involved evaluation of muscle strength by two physical therapists in 29 patients ventilated >48 hours. Manual strength testing was performed by both physical therapists using two techniques: isometric and through range; and two scoring systems: traditional six-point Medical Research Council scale and a new collapsed four-point scale. Part two involved assessment of handgrip strength conducted on 60 patients. A cutoff score for ICU-AW was identified for the new four-point scoring system.

**Results:**

The incidence of ICU-AW was 42% (n = 25/60) in this study (based on HGD). In part one the highest reliability and agreement was observed for the isometric technique using the four-point scale (intraclass correlation coefficient = 0.90: kappa = 0.72 respectively). Differences existed between isometric and through-range scores (mean difference = 1.76 points, *P* = 0.005). In part two, HGD had a sensitivity of 0.88 and specificity of 0.80 for diagnosing ICU-AW. A cutoff score of 24 out of 36 points was identified for the four-point scale.

**Conclusions:**

The isometric technique is recommended with reporting on a collapsed four-point scale. Because HGD is easy to perform and sensitive, we recommend a new two-tier approach to diagnosing ICU-AW that first tests handgrip strength with follow-up strength assessment using the isometric technique for muscle strength testing if handgrip strength falls below cutoff scores. Whilst our results for the four-point scale are encouraging, further research is required to confirm the findings of this study and determine the validity of the four-point scoring system and cutoff score developed of less than 24 out of 36 before recommending adoption into clinical practice.

**Electronic supplementary material:**

The online version of this article (doi:10.1186/s13054-015-0780-5) contains supplementary material, which is available to authorized users.

## Introduction

Intensive care unit-acquired weakness (ICU-AW) is a significant and prevalent problem for individuals who survive the initial insult of a critical illness [1,2]. Timely and accurate diagnosis is important in order for clinicians to target rehabilitation resources and select appropriate exercise modalities to minimise further muscle wasting [3]. It is important that muscle strength testing for diagnosis is reliable, valid and easily performed by different members of the multidisciplinary team.

Manual muscle testing (MMT) using the six-point Medical Research Council sum score (MRC-SS) is currently the recommended method for diagnosing ICU-AW [4-6]. There is criticism within the literature of this scoring system, particularly in its ability to discriminate between strength categories at the upper end of the scale (for example, between Grades 4 to 5 on the Oxford grading system) [7-9]. Whilst there is high inter-rater reliability for MRC-SS, the agreement levels reported for diagnosing ICU-AW have varied from poor to good in previous studies [8,10-13]. Worldwide there is variability in the testing technique utilised by clinicians, as strength testing can be assessed either isometrically (at one point in the range) or whilst moving through a joint’s range of motion [14].

Recently, a modified four-point scoring system with a transformed maximum sum score of 36 was developed in an attempt to improve the MRC grading system accuracy and to restore the ordered threshold requirements for ordinal categories [15]. This four-point scale was derived from the original six-point scoring system for muscle strength testing. The validity of this new scoring system has not been established in the intensive care unit (ICU) setting.

Thorough MMT requires clinicians to be adequately trained in the assessment procedures and it can take up to 30 minutes to test six muscle groups bilaterally. To reduce the time taken for assessment, handgrip dynamometry (HGD) has been recommended as a simple and easy surrogate measure for diagnosing ICU-AW [13]. Ali and colleagues developed cutoff scores for HGD based on gender (for males <11 kilograms (kg) and for females <7 kg is considered to be indicative of ICU-AW) [13]. However, uptake of HGD as a diagnostic assessment tool in clinical practice has been inconsistent with no published external validation of these previously developed cutoff scores.

Therefore, this study aimed to examine the methodological practices for MMT and HGD to provide a simplified standard recommendation for the clinical diagnosis of ICU-AW. This study was conducted in two parts. Parts of the results from this study have been reported in abstract format [16]. The CONsensus-based Standards for the selection of health Measurement INstruments (COSMIN) guidelines [17] were followed in the reporting of the evaluation of the reliability and validity of measures.

The primary aims of part one were to: (1) determine the inter-rater reliability for MMT and agreement for the diagnosis of ICU-AW using the six-point MRC-SS and the new four-point scoring systems; (2) examine differences between isometric and through-range technique and (3) determine the inter-rater reliability for HGD strength assessment. The primary aim of part two was to: (1) establish the validity of handgrip dynamometry and determine a cutoff point using the four-point scoring system for diagnosis of ICU-AW.

## Materials and methods

### Study design and setting

This is a single-centre prospective study performed at a quaternary mixed medical and surgical ICU in Melbourne, Australia. The Austin Health institutional ethics committee approved the study. Written informed consent was not required from the patients for participation in this study as it involved the analysis of routinely collected data. Therapists provided verbal consent to the participation in this study.

### Assessors

Part one (reliability) involved two ICU physical therapists who evaluated a sample of 29 patients over a seven-month period between May and November 2012. Part two (validity) involved eight physical therapists of differing levels of expertise and took place from May 2012 to August 2013. Patients assessed in part one were included in the overall analyses performed within part two to provide data on an overall sample of 60 patients in order to enable an informed recommendation for a new two-tier approach to the clinical diagnosis of ICU-AW. The assessors were a convenience sample of physical therapists who were involved in the routine care of patients with critical illness at this centre over the study period. All assessors received training in MMT and HGD and a standardised protocol was followed at all times.

### Study procedures

Data were collected on strength assessments performed by physiotherapists on adult patients (>18 years) with critical illness who were mechanically ventilated for more than 48 hours. Strength measurements were performed on the day of awakening. This was defined as the first day that the patient was alert with a Riker sedation agitation scale score between three and five [18] and ability to follow at least three of the De Jonghe five-command criteria [2]. Whilst no reliability or validity has been established for these five-point criteria, they have extensively used to evaluate awakening in the critical care research literature [2,10,19].

In part one (reliability), two physical therapists independently conducted MMT within a 24-hour period. MMT was evaluated using both techniques (isometric and through range) and scored using two scales (six-point [5] and four-point [15]) (Table 1). Four separate testing sessions were conducted (two per assessor) to enable determination of the reliability of different techniques and scoring systems. A 24-hour period in which all tests needed to be performed was chosen as a change in score clinically due to patient recovery would not be expected in this time frame, and thus should not influence the results obtained by the different assessors. In part two (validity) only the isometric technique was adopted based on the findings within part one and results were scored using both scales (six-point and four-point) (Table 1).

**Table 1:** **Medical research council sum score: six-point and four-point ordinal scales for assessment**

**Six-point ordinal scale**	**Four-point ordinal scale**
0 = no muscle contraction	0 = paralysis
1 = flicker or trace of muscle contraction	1 = severe weakness defined as >50% loss of strength
2 = active movement with gravity eliminated	
3 = reduced power but active movement against gravity	2 = slight weakness <50% loss of strength
4 = reduced power but active movement against gravity and resistance	
5 = normal power against full resistance	3 = normal strength

Screening for awakening and comprehension were evaluated on each testing occasion by each physical therapist. Both physical therapists assessed each patient in the same position in bed. At the first testing time point, assessor order and technique (isometric or through range) were randomly assigned by independent personnel not involved in the study using a random number generator and sealed opaque consecutively numbered envelopes. All assessments were performed within a 24-hour period, which enabled adequate rest in between assessments to minimise patient fatigue. Assessors were blinded to each other’s measurements. Patients were stable throughout the 24-hour testing period and testing conditions were similar at all four testing time points.

### Manual muscle strength testing

Six muscle groups bilaterally were evaluated (shoulder abduction, elbow flexion, wrist extension, hip flexion, knee extension and ankle dorsiflexion). For the traditional six-point scoring system a score of less than 48 out of 60 is considered indicative of ICU-AW [2]. Muscle strength was initially assessed against gravity; if the patient was unable to perform the movement against gravity then the position was modified. In part one (reliability) MMT was evaluated both isometrically and through range. An isometric hold is when the resistance is applied at one point only and the subject pushes against this resistance. There is no movement of the joint. For example for shoulder abduction the assessor would apply resistance at 90 degrees shoulder abduction (mid range). Through-range testing means that the assessor provides resistance to the muscle whilst the joint is moving. For example, for shoulder abduction the assessor would apply resistance with the subject’s arm by their side at 0 degrees and continue to apply resistance until the patient reaches past 90 degrees abduction. The isometric technique has been well described previously in the literature [20,21]. Through-range testing was performed in a standardised manner through the joint’s range of motion. Therapist hand positioning and patient positioning were similar to that described in the isometric methodology for the through-range technique. Table S1 in Additional file 1 contains detailed description of the differences between isometric and through-range testing for each movement assessed.

Two scoring systems were utilised to quantify muscle strength using MMT. The traditional six-point scoring system is a six-point scale ranging from 0 = no muscle contraction to 5 = normal power against full resistance as shown in Table 1. The modified (four-point) scoring system has four scores (0 = paralysis, 1 = severe weakness >50% loss, 2 = slight weakness <50% loss, and 3 = normal strength) as shown in Table 1. This scoring system restores the ordered threshold requirements for ordinal categories [15]. It has been demonstrated that there is poorer agreement between assessors at the higher end of the six-point scoring system particularly in discriminating between 4 (reduced power) and 5 (normal strength) [7-9].

### Handgrip dynamometry

A Jamar hydraulic dynamometer (Sammons Preston Rolyan, Bolingbrook, IL, USA) was used to evaluate handgrip strength. Prior to commencing this study two dynamometers were calibrated. The same two dynamometers were used throughout the study and manufacturer specifications for equipment care and storage were followed. The physiotherapist evaluated HGD if the patient had at least antigravity strength for both elbow flexor and wrist extensor muscle groups. Handgrip dynamometry was measured immediately post MMT on all occasions. Standardised instructions and encouragement were provided to patients by the physical therapist. In order to facilitate effective blinding of the physiotherapists, patients were not told the results of their efforts. The instructions during testing were similar to that previously described in the ICU literature [22] and patient set-up for assessment of HGD was conducted according to established guidelines for HGD testing [23]. Patients were given at least six seconds to generate maximum peak force, with a minimum of 60 seconds rest in between each test [22]. The highest HGD scores recorded (right and left) of three attempts were used in final analyses. In part two the cutoff scores previously developed by Ali and colleagues were used to classify presence or absence of ICU-AW based on gender [13]. The cutoff scores are <11 kg for males and <7 kg for females [13]. The overall incidence of ICU-AW was calculated based on the cutoff scores previously developed by Ali and colleagues. Handgrip strength was assessed in both part 1 (reliability) and part 2 (validity). MMT was utilised as the standard reference in part two (using the traditional six-point scoring system and isometric technique) against which HGD was compared for determining the validity and test performance of the previously developed HGD cutoff scores [13]. Although manual muscle strength testing has its limitations, it is currently recommended as the main diagnostic method for identifying ICU-AW at this point in time [24].

### Other information collected

Baseline demographic information on admission diagnosis, gender, age, mechanical ventilation time, severity of illness according to the Acute Physiology and Chronic Health Evaluation Two (APACHE II) score were collected for the patients assessed in this study. Time to awakening, Physical Function in Intensive Care Test (PFIT) scores on awakening and ICU discharge, ICU and hospital length of stay (LOS), discharge destination were also recorded. Baseline demographic information (age, gender, ICU clinical experience in years) was also obtained from the physiotherapists participating in this study.

### Statistical power and analyses

We calculated that 24 patient assessments were required to achieve a reliability coefficient >0.8 and *P* <0.05, in part one (reliability) [25]. Additionally, we calculated that 60 patients were required to test the sensitivity and specificity (chi-square analyses) of the four-point scale. In part two (validity) the sample size required was determined based on grip strength and four-point scoring system sensitivity and specificity and receiver operating characteristic (ROC) curve analyses. A sensitivity of 0.75 and specificity of 0.70 with an area under the curve (AUC) characteristic of 0.80 was determined *a priori* as acceptable. A score above 0.80 for AUC is considered good, and scores above 0.90 excellent [26]. Using these values we calculated that a sample size of 60 patients were required ([27] (accessed May 2012)).

In part one (reliability), both intraclass correlation coefficients (ICCs) (2, 1) and weighted linear kappa statistics were calculated to determine inter-rater reliability and agreement for diagnosing ICU-AW respectively. Paired *t* tests and Bland-Altman analysis were utilised to determine mean differences between isometric and through-range techniques. In part two (validity), chi-square analyses and the ROC curve were used to determine the test performance of HGD testing and to identify a cutoff score on the four-point scale, which would have the highest sensitivity and specificity for diagnosing ICU-AW. Convergent validity was examined using correlations (rho) for MMT, HGD, PFIT score, discharge destination, hospital parameters; mechanical ventilation time and ICU and hospital LOS. Parametric data were presented as mean and standard deviation (SD), and non-parametric data as median and interquartile range [IQR]. An alpha value <0.05 was considered statistically significant. SPSS for Macintosh statistical software package (SPSS Statistics version 20.0; IBM Corp, Armonk, NY, USA) was used in statistical analyses.

## Results

In part one (reliability), the assessors were two female physical therapists with five and eight years of clinical experience respectively in rehabilitation and strength testing in the ICU setting. In part two, eight physical therapists (all female) were involved. Levels of clinical experience ranged from six months to eight years, and ICU-specific clinical experience ranged from four months to five years.

Table 2 describes the demographic characteristics of the sample of 60 patients on whom assessments of muscle strength were performed. This sample includes the 29 patients tested within part one*.* The overall incidence of ICU-AW in this cohort was 42% (n = 25/60) based on handgrip dynamometry scores and their median [IQR] APACHE II score was 22 [17-22,24-26,28,29]. All physiotherapists were able to conduct a thorough strength examination including both MMT and HGD; there were no missing data from final analyses.

**Table 2:** **Demographics of the patients evaluated by the physical therapists (n = 60)**

**Characteristic**	**Total cohort n (%) or median [IQR]**
Male	35 (58%)
Age, years	69 [49–77]
MV time, hours	159 [89–294]
APACHE II	22 [18-29]
Admission category	
Medical	28 (47%)
Surgical	24 (40%)
Other	8 (13%)
Awakening time, days	9 [5-12]
Total ICU LOS, days	12 [8-20]
Total hospital LOS, days	25 [18–41]
Overall in-hospital mortality	8 (13%)

n, number; IQR interquartile range; MV, mechanical ventilation; APACHE II, Acute Physiology and Chronic Health Evaluation II; ICU, intensive care unit; LOS, length of stay.

### Part one – reliability

Inter-rater reliability for the overall scoring of manual muscle strength according to the MRC-SS was excellent regardless of the testing technique or scoring system utilised as represented by the ICCs shown in Table 3. However, the strength of agreement for diagnosis of ICU-AW (less than 48 out of 60) was more variable with only fair agreement (kappa = 0.26) for the through-range technique using the six-point scale versus substantial agreement for the isometric technique using the six-point scale (kappa = 0.72) (Table 3).

**Table 3:** **Inter-observer agreement for testing method and scoring system (manual muscle strength testing and handgrip dynamometry (n = 29))**

**Testing procedure**	**Inter-rater reliability ICC [95%CI]**	**Kappa agreement for diagnosis of ICU-AW**
Six-point isometric	0.88 [0.75-0.94]	0.72^a^
Six-point through range	0.78 [0.55-0.90]	0.26^a^
Four-point isometric	0.90 [0.80-0.95]	0.85^b^
Four-point through range	0.94 [0.87-0.97]	0.63^b^
Overall cohort Right HGD	0.93 [0.85-0.97]	
Overall cohort Left HGD	0.98 [0.95-0.99]	
Females Right HGD	0.97 [0.90-0.99]	
Females Left HGD	0.94 [0.82-0.98]	
Males Right HGD	0.88 [0.70-0.96]	
Males Left HGD	0.97 [0.91-0.99]	

^a^Kappa statistic using binary outcome of clinical weakness for six-point scale (less than 48 out of 60); ^b^kappa statistic using binary outcome of clinical weakness for four-point scale (less than 24 out of 36). n, number; ICC, interclass correlation coefficient; 95%CI, ninety-five percent confidence interval; ICU-AW, intensive care unit-acquired weakness; HGD, handgrip dynamometry.

There was a significant difference between isometric and through-range techniques when using the traditional six-point scoring system (mean difference = 1.76 points (out of 60), ninety-five percent confidence interval (95%CI) = 0.58 to 2.94, *P* = 0.001). The mean ± SD MRC-SS out of 60 was 48 ± 6 for the isometric technique and 46 ± 8 for through-range technique using the six-point scale. The mean ± SD MRC-SS out of 36 was 27 ± 6 for the isometric technique and 26 ± 6 for the through-range technique using the new collapsed four-point scale.

There was also a significant difference between assessors in terms of mean MRC-SS with the six-point scale when using the through-range technique (Table 4). Bland-Altman analysis demonstrated smaller mean differences and narrower limits of agreement for the four-point scoring system compared to the six-point scoring system as shown in Table 4. The through-range six-point testing technique had the greatest mean difference at 2.55 points with wider limits of agreement compared to the other three testing combinations (Table 4).

**Table 4:** **MRC-SS Bland-Altman results from part one (n = 29)**

**Testing procedure**	**MRC-SS mean ± SD**		**ICU-AW incidence n (%)**					
	**Assessor 1**	**Assessor 2**	**Assessor 1**	**Assessor 2**	***P*** **value**	**Mean diff**	**LOA (+)**	**LOA (−)**
Isometric six-point	48 ± 9	48 ± 9	12 (41%)	14 (48%)	0.74	−0.27	9.04	8.48
Through range six-point	48 ± 8	45 ± 9	15 (52%)	17 (59%)	0.02	2.55	12.99	7.88
Through range four-point	26 ± 6	26 ± 6	9 (31%)	12 (41%)	0.23	0.48	4.62	3.66
Isometric four-point	27 ± 6	27 ± 6	10 (35%)	10 (35%)	0.85	−0.10	5.43	5.63

MRC-SS, Medical Research Council sum score; SD, standard deviation; ICU-AW, intensive care unit-acquired weakness; n, number; mean diff, mean difference; LOA, limit of agreement; *+*, positive; *−*, negative.

The inter-rater reliability for HGD testing between two physical therapists was almost perfect for the overall cohort including both left and right handgrip scores (Table 3). The reliability of HGD was slightly lower for males (although not statistically significant) than for females when examining right HGD scores as shown in Table 3.

### Part two – validation and recommendation of a standard diagnostic approach

Based on the findings of part one (reliability), in part two (validity) the isometric technique was adopted*.* The median [IQR] for MRC-SS was 48 [41 to 53] out of 60 with scores ranging from 10 to 60.

### Validation of HGD as a surrogate measure for diagnosis of ICU-AW

The accuracy of HGD scores in the diagnosis of ICU-AW was compared to the traditional MRC six-point score (less than 48 out of 60). The sensitivity and specificity of HGD as a surrogate measure for ICU-AW was high and clinically acceptable overall with similar results for both right and left sides (Table S2 in Additional file 2). For females, specificity was poorest (specificity = 0.45 to 0.55), however sensitivity was perfect (sensitivity = 1.0) (Table S2 in Additional file 2).

The median [IQR] HGD score for the cohort was 10.5 [0 to 21.5] kg. Based on gender the median [IQR] HGD scores were for females: 0 [0 to 7.3] kg and for males: 20 [10 to 40] kg. Twenty-seven percent (n = 16/60) of patients assessed had a handgrip score of zero, with the majority of those who scored zero being female (n = 14/16, 88%). All but three patients (with grip score of zero) had a clinical diagnosis of ICU-AW based on MRC sum score (less than 48 out of 60). There was no correlation between age and HGD score (rho = 0.131, *P* = 0.320). Six individuals scored zero, and had no antigravity strength in their elbow and/or wrist, and all six were classified with severe ICU-AW (less than 36 out of 60) based on the traditional six-point scoring system. Convergent validity was established with a significant large correlation between HGD score and the six-point scoring diagnosis of ICU-AW (rho = 0.86; *P* <0.001) and awakening PFIT score (rho = 0.56; *P* <0.001). Significant moderate correlations were identified for HGD and mechanical ventilation hours (rho = −0.30; *P* = 0.02); hospital LOS (rho = −0.30; *P* = 0.002); discharge PFIT score (rho = 0.38; *P* = 0.004) and discharge to home (rho = −0.36; *P* <0.001).

### Validating the four-point scoring system for MMT

**Figure 1:**
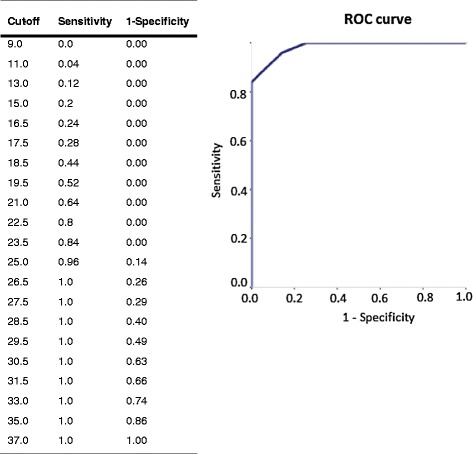
**Determining cutoff score for the four-point scoring system from coordinates of the receiver operating curve for highest sensitivity and specificity.** The graph on the right is called a receiver operating characteristic curve (ROC curve). It is a plot of the true positive rate (y-axis) against the false positive rate (x-axis) for the different possible cut-points of a diagnostic test. The closer the curve is to the left-hand border and top border of the ROC space the more accurate the test. Accuracy is measured by the area under the curve. An area of 1 = perfect test; an area of 0.5 = inadequate test. The ROC curve analysis resulted in an area under the curve of 0.92 (95%CI 0.83 to 1.0), which is almost perfect and demonstrates excellent diagnostic accuracy. The table on the left outlines each individual plotted cut-point. At 23.5 the sensitivity was 0.84, with specificity of 1.0, and at 25 the sensitivity was 0.96, and specificity was 0.86. A cutoff point of 24 would therefore result in high sensitivity and specificity. 95%CI, ninety-five percent confidence interval.

A cutoff score of 24 out of 36 was identified for the four-point scoring system (Figure 1) using an isometric testing procedure with excellent discriminative ability (area under the ROC curve (95%CI) = 0.92 (0.83 to 1.0) and excellent diagnostic accuracy (sensitivity (95%CI) = 0.84 (0.64 to 0.96); specificity (95%CI) = 1.0 (0.9 to 1.0); positive predictive value (95%CI) = 1.0 (0.84 to 1.0); and negative predictive value (95%CI) = 0.90 (0.76 to 0.98)).

Convergent validity was identified with a significant large correlation between the four-point scoring system for diagnosis of ICU-AW (less than 24 out of 36) with awakening PFIT score (rho = 0.70; *P* <0.001) and handgrip strength (rho = 0.66, *P* <0.001). A significant moderate correlation was identified between the four-point scoring system for diagnosis of ICU-AW (less than 24 out of 36) and mechanical ventilation hours (rho = −0.42; *P* = 0.001); ICU LOS (rho = −0.39; *P* = 0.01); time to awakening (rho = −0.39; *P* = 0.002); hospital LOS (rho = 0.45; *P* <0.001); and discharge to home (rho = −0.48; *P* <0.001).

## Discussion

The findings of this study have important implications for clinicians in the diagnosis of ICU-AW using volitional strength testing at the bedside. First, we found that there was excellent inter-rater reliability for overall MRC-SS regardless of testing technique or scoring system utilised. However, there was a significant difference between the use of isometric and through-range techniques. There were also greater mean differences between both assessors when using the through-range technique and the inter-rater agreement for the diagnosis of ICU-AW was less accurate (kappa = 0.26) using the through-range technique compared to the isometric technique (kappa = 0.72). Based on these findings from part one (reliability), the isometric technique is the preferred method for the evaluation of MMT in ICU. In part two (validity) a cutoff score for the four-point scoring system was identified as less than 24 out of 36 for the diagnosis of ICU-AW. Similar to the six-point scoring system, we identified that greater levels of agreement existed for the diagnosis of ICU-AW when using an isometric technique. Therefore, the four-point scoring system may provide greater inter-rater agreement between assessors in the quantification of muscle strength. Our study also demonstrated that HGD is both a highly reliable and valid measurement tool for the screening of ICU-AW with excellent test performance on this external validation.

Manual muscle strength testing is time-consuming and requires expertise and training to administer appropriately [14,20]. In contrast, HGD is a simple quick tool, which can be assessed with limited training in a few minutes to screen for the presence of ICU-AW, and can be incorporated easily into daily assessment practices. Service provision of therapy varies from institution to institution with many facilities not having a designated physical therapist on staff. It is also important to note that not everyone who is in ICU will develop ICU-AW. Therefore, using HGD that is simple and can be performed by any multidisciplinary member is a feasible and valid option for initial ICU-AW screening.

The incidence of ICU-AW varies across different settings but is reported to be around 25 to 50% in the general ICU setting across several studies [2,10,19]. The true incidence of ICU-AW has been challenging to elucidate due to the variability and inconsistencies in testing methodology, which have limited the generalisability and comparability of findings between studies. This study, therefore, addresses some of these inconsistencies and provides a standardised approach to the assessment of manual muscle strength testing. This study demonstrated that the isometric technique is superior to through-range measures in terms of agreement between assessors for classifying the presence or absence of ICU-AW regardless of the scoring system used. This may be due to differences in factors such as deceleration, acceleration and changes in the mechanical advantage of the limb during the through-range technique [28].

The six-point MRC-SS has been used for more than a decade as a diagnostic tool for the identification of ICU-AW [2,5]. Despite its widespread use there are several methodological shortcomings with this scale. Previous research has highlighted that there are greater discrepancies in scoring between assessors at higher grades (greater than Grade 3 - antigravity strength) [8]. Our study demonstrated that the new collapsed four-point scoring system with a cutoff score of less than 24 out of 36 has both excellent reliability and agreement for the diagnosis of ICU-AW between assessors compared to the six-point scoring system. The validity of the four-point scoring system was also demonstrated with significant correlations to measures of physical function, strength and parameters such as mechanical ventilation time and LOS.

The research into the four-point scale is in its embryonic development. There are two potential advantages of the four-point scale over the traditional six-point scale method. First, the collapsed four-point scale restores weighting between levels based on Rasch analytical principles as described by Vanhouette and colleagues [15,29]. The six-point scoring system and sum score out of 60 are ordinal based and suggest equal weighting at each grade, which is not the case [15,29]. Concern has been raised that this disordered threshold impacts on the accuracy of results [15,29]. A recent study (albeit not specifically in the ICU setting) demonstrated 80% of all muscles examined were incorrectly classified [15]. The greatest inconsistencies were observed for Grades 2 to 4 [15]. Within the ICU population, a recent study demonstrated variable agreement for individual muscle groups ranging from 35 to 75% between assessors [10]. Some studies within the ICU have demonstrated that the greatest challenge was in the differentiation between scores 4 and 5 [8]. To improve clinical applicability based on Rasch analytical principles and to restore the weighting to scores a four-point score was developed by Vanhouette and colleagues [15,29].

Another possible advantage of the four-point scale is that it could potentially be used by less experienced clinicians as there is less discrimination between grades required. Concerns on the potential subjectivity of the four-point scale have been raised within the literature [30]. This study presents data for the first time on the reproducibility and potential clinical relevance of the four-point scale for diagnosis of ICU-AW. Whilst our findings are promising, more work needs to be performed to examine the four-point scale before it is recommended/adopted into clinical practice.

For HGD our study demonstrated high sensitivity, specificity and predictive values that were similar to those reported by Ali and colleagues [13], which indicates that the scores maintained stability in an independent sample validation. A recent study examined HGD in a surgical ICU setting and suggested that handgrip strength had a significant floor effect with 55% of the cohort scoring zero [31]. Some individuals had a score of zero and had acceptable or normal manual muscle strength test scores [31]. It is important to note there were differences between studies in terms of screening for alertness and comprehension and also more importantly differences in HGD hold time to allow peak muscle contraction to be reached. Baldwin and colleagues found that critically ill individuals require at least six seconds to generate their peak force [22], which is twice the length of time that was applied by Lee and colleagues in their study [31]. Therefore, it is possible in the study by Lee and colleagues that patients were not given sufficient time to reach their peak muscle force levels [31].

In this study there was no correlation between age and HGD score. Based on the findings of this study and previous research, the handgrip cutoff scores have been shown to be sensitive and able to diagnose the presence of ICU-AW based on gender [13]. Normative data on handgrip dynamometry are often stratified by gender and also age [23,32]. Whether cutoff scores need to be considered particularly for younger individuals based on age as well as gender could be considered in the future. This may be more important when monitoring patient recovery over time in order to be able to compare to normative age/gender-matched data, rather than for the diagnosis of ICU-AW.

In our study, a floor effect was observed with strength testing using HGD with 30% scoring zero on testing, which is similar to the floor effect reported by Ali and colleagues with 26% of their cohort scoring between zero and five kilograms [13]. Although there may be a floor effect with HGD testing, the majority of patients who scored zero had a diagnosis of ICU-AW, and perhaps if used as a first tier of screening for the presence of ICU-AW it may inform the therapist that further thorough assessment of manual muscle strength testing is required. In females the sensitivity for HGD was perfect (1.0) and specificity was lower (0.45 to 0.55). A lower specificity means that there are individuals who are diagnosed with ICU-AW based on handgrip scores who on thorough manual muscle strength testing would not be identified with ICU-AW. It would be more of a concern if sensitivity were low, as this would mean that individuals who had ICU-AW would be missed, and this may have significant clinical implications for the management of the patient.

### Limitations

The key limitation with this study is within part one (reliability) where there is the possibility of recall bias, as the physical therapist could not be blinded from the results of their previous testing session and that only two assessors were included. In part two (validity), the assessors had a varying range of expertise both as physical therapists and also specifically in terms of practical expertise within the ICU setting, however this may improve the generalisability of the findings. Other limitations include: small sample size and single-centre study design.

There are inherent limitations with volitional muscle strength testing using HGD and MMT. Testing requires patients to be awake and co-operative. The feasibility of strength testing in our study was limited with 15% (n = 42/283) unable to be assessed due to inattention during their ICU stay, which is consistent with the findings of previous studies [8,10]. The median [IQR] time to awakening in our study was 9 [5-11] days. This is consistent with previous studies, which reported a delay of 10 to 18 days [8,10]. These are important limitations of volitional strength testing in general.

### Future directions

Studying non-volitional clinical measures in the future, which can easily be evaluated at the bedside on admission to identify those at risk of ICU-AW, is warranted. Whilst modalities such as neuromuscular ultrasound imaging are being investigated [33,34] the current method for screening and diagnosing ICU-AW will continue to be clinical strength testing.

This study is focused on developing a standard simple diagnostic screening approach to identify ICU-AW. It is important to note that there is inconclusive evidence to support the use of MMT or HGD to measure change over time in order to evaluate treatment efficacy or recovery post critical illness with one study suggesting that at least a 50% change in muscle strength score from baseline is required to reflect a true change [22]. Further research is required to determine what outcome measure/s should be utilised to monitor and measure intervention efficacy across the continuum of ICU recovery. Future research also warrants investigation of strength measures such as HGD and MMT against electrophysiological testing in order to understand further the changes in muscular control and strength generation.

## Conclusions

The findings of this study informed the development of a new two-tier approach to screening for the presence of ICU-AW on awakening. The first tier involves assessment of handgrip strength (with a score <11 kg for males and <7 kg for females indicative of ICU-AW). Handgrip dynamometry is quick and easily administered with minimal training by any member of the multidisciplinary team. This is particularly advantageous in units where there may be limited access to physical therapists or other rehabilitation staff, as it will facilitate early identification of individuals who may benefit from therapy. If patients fall below the cutoff levels on HGD testing or are unable to perform HGD (due to lacking antigravity strength in elbow flexors/wrist extensors), a referral to a therapist is warranted to enable a more thorough strength assessment to be conducted. Manual muscle strength testing should then be assessed using an isometric technique. Further research is required to confirm the findings of this study and determine the validity of the four-point scoring system and cutoff score developed (less than 24 out of 36) before recommending adoption into clinical practice.

In the future, a third tier may be warranted where nerve conduction testing is performed to gather further information to identify neuropathy as opposed to myopathy [35]. Further research into the phenotype of muscle weakness is required. These methods will enable physical therapists and rehabilitation staff to target rehabilitation resources and select appropriate exercise modalities to minimise muscle wasting and improve the longer-term outcomes for the survivors of critical illness who will most benefit.

## Key messages

- There is a difference between isometric and through-range techniques for assessing manual muscle strength and the isometric technique is the preferred technique for assessing manual muscle strength (with higher accuracy and reliability).
- A cutoff score of 24 out of 36 was identified for the four-point scoring system.
- Handgrip dynamometry is a valid and reliable surrogate tool for diagnosing ICU-AW.
- Two-tier approach to muscle strength testing is recommended (1) handgrip testing and (2) thorough manual muscle strength testing if below the handgrip cutoff levels.

## Additional files

Additional file 1: Table S1. Methodology description for through-range and isometric testing. Additional file 2: Table S2. Individuals with a diagnosis of ICU-AW as determined by handgrip dynamometry.

## Abbreviations

95%CI: ninety-five percent confidence interval
APACHE II: Acute Physiology and Chronic Health Evaluation Two
AUC: area under the curve
COSMIN: CONsensus-based Standards for the selection of health Measurement INstruments
HGD: handgrip dynamometry
ICC: intraclass correlation coefficient
ICU: intensive care unit
ICU-AW: intensive care unit-acquired weakness
IQR: interquartile range
LOS: length of stay
MMT: manual muscle testing
MRC-SS: Medical Research Council sum score
n: number
PFIT: Physical Function in Intensive Care test
ROC: receiver operating characteristic
SD: standard deviation
