# Supplementary material for: A new two-tier strength assessment approach to the diagnosis of weakness in intensive care: an observational study
Source: Crit Care. 2015 Feb 26;19(1):52. doi: 10.1186/s13054-015-0780-5 (PMC4344764; doi:10.1186/s13054-015-0780-5)
Supplement: Additional file 1: Table S1. — Methodology description for through-range and isometric testing. [file 13054_2015_780_MOESM1_ESM.docx]

Additional file 1: Table S1: Methodology description for through range and isometric testing

| **Movement assessed** | **Therapist Positioning** | **Through range testing description** | **Isometric testing description** |
| --- | --- | --- | --- |
| **Shoulder abduction** | Therapist applies resistance above the elbow with other hand stabilising over the shoulder region | Resistance applied as subject moves from full shoulder adduction up to 90 degrees shoulder abduction | Resistance applied at 90 degrees shoulder abduction |
| **Elbow flexion** | Therapist applies resistance over the flexor surface of the forearm with other hand stabilising over the anterior shoulder region | Forearm supinated and resistance applied as subject moves from full elbow extension through to around 110 degrees elbow flexion | Resistance applied at around 110 degrees elbow flexion |
| **Wrist extension** | Therapist applies resistance over dorsal surface of the hand distal to the wrist joint with other hand stabilising above the wrist joint | Arm by side elbow flexion to 90 degrees with forearm pronated. Resistance applied as subject moves from neutral through to full wrist extension (fingers flexed) | Resistance applied at full wrist extension (fingers flexed). |
| **Hip flexion** | Therapist applies resistance over anterior thigh proximal to the knee joint with other hand stabilising over lateral hip joint region | Resistance applied as subject moves from 90° hip flexion through the full hip flexion range | Resistance applied at 90 degrees hip flexion |
| **Knee extension** | Therapist applies resistance proximal to the ankle joint. Patient tested in supine, a 5 inch fulcrum roll is placed under the knee. | Resistance is applied as subject moves from knee flexed through to full extension | Resistance is applied at full knee extension only. |
| **Ankle dorsiflexion** | Therapist applies resistance over dorsal surface of the foot and other hand stabilises above the ankle joint | Resistance applied as subject moves from plantigrade through to full dorsiflexion | Resistance applied at full dorsiflexion |
