# Supplementary material for: A new two-tier strength assessment approach to the diagnosis of weakness in intensive care: an observational study
Source: Crit Care. 2015 Feb 26;19(1):52. doi: 10.1186/s13054-015-0780-5 (PMC4344764; doi:10.1186/s13054-015-0780-5)
Supplement: Additional file 2: Table S2. — Individuals with a diagnosis of ICU-AW as determined by handgrip dynamometry. [file 13054_2015_780_MOESM2_ESM.docx]

Additional file 2: Table S2: Individuals with a diagnosis of ICU-AW as determined by handgrip dynamometry

| **Group** | **Hand side** | **AUC** | **Sensitivity** | **Specificity** | **PPV** | **NPV** |
| --- | --- | --- | --- | --- | --- | --- |
| Overall | Right | 0.84 | 0.88 | 0.80 | 0.76 | 0.91 |
|  | Left | 0.85 | 0.96 | 0.74 | 0.72 | 0.96 |
| Females | Right | 0.77 | 1.0 | 0.55 | 0.74 | 1.0 |
|  | Left | 0.73 | 1.0 | 0.45 | 0.70 | 1.0 |
| Males | Right | 0.82 | 0.91 | 0.88 | 0.77 | 0.95 |
|  | Left | 0.89 | 0.72 | 0.92 | 0.80 | 0.88 |

***Abbreviations:*** *AUC, area under the curve; ICU-AW, intensive care unit acquired weakness; NPV, negative predictive value; PPV, positive predictive value*
